# Chronic Post-Traumatic Diaphragmatic Hernias: Diagnostic Pitfalls, Surgical Strategies, and Long-Term Outcomes

**DOI:** 10.7759/cureus.95504

**Published:** 2025-10-27

**Authors:** José Emiliano González Flores, Pablo Orozco Obregón, José P Orozco Hidalgo, Airam A. Arias Villaverde, Emiliano Murillo Mendoza, Ana D Zamudio Carías, Esmeralda Y Galán Dávalos, Pablo Navarro López, Alfonso Sandoval, Luis M Canal de Velasco, Nathalia García Martínez, Emilio Mondragón Rosas

**Affiliations:** 1 Surgery, Tecnológico de Monterrey Campus Ciudad de Mexico, Mexico City, MEX; 2 Surgery, American British Cowdray Medical Center, Mexico City, MEX; 3 School of Medicine and Health Sciences, Tecnológico de Monterrey Campus Ciudad de Mexico, Mexico City, MEX; 4 School of Medicine and Health Sciences, Tecnológico de Monterrey Campus Ciudad de Mexico, CDMX, MEX; 5 Medical Education and Simulation, Panamerican University, Mexico City, MEX

**Keywords:** chronic post-traumatic diaphragmatic hernia (cpdh), contrast-enhanced ct, delayed diagnosis, mesh reinforcement, minimally invasive surgery, surgical repair

## Abstract

Chronic post-traumatic diaphragmatic hernias (CPDH) represent uncommon clinical entities that frequently remain undiagnosed due to their wide spectrum of clinical manifestations, ranging from asymptomatic presentations to severe complications. This review explores the diagnostic difficulties, current surgical options, and long-term outcomes associated with CPDH, while highlighting the absence of consensus-based management guidelines. A review of eight published case series was performed to evaluate demographic characteristics, diagnostic delays, operative approaches, mesh use, postoperative outcomes, and recurrence rates. The findings show that symptoms may develop months or years after the initial trauma, with contrast-enhanced CT emerging as the most reliable diagnostic modality despite frequent delays in detection. Minimally invasive approaches, including laparoscopic and robotic techniques, are increasingly preferred in elective cases given their association with lower morbidity, although the choice of repair strategy and mesh material remains variable and individualized. This review emphasizes the need for heightened clinical awareness, development of standardized protocols, and further research focused on long-term outcomes and recurrence prevention.

## Introduction and background

Chronic post-traumatic diaphragmatic hernias (CPDH) are uncommon but clinically significant entities that often remain undiagnosed because of their variable and nonspecific presentation. They occur when abdominal contents migrate into the thoracic cavity through a diaphragmatic defect, most commonly after blunt or penetrating trauma, although iatrogenic cases following major abdominal surgery have also been reported [1-3]. Unlike congenital diaphragmatic hernias, which predominate in pediatric populations, CPDH in adults are acquired forms that may stay clinically silent until organ compromise produces respiratory or gastrointestinal symptoms, or even life-threatening complications such as strangulation or respiratory failure [4,5].

Diagnosis is frequently delayed. Early manifestations are subtle, and standard imaging may be normal in a substantial proportion of patients, leading to missed recognition during the acute phase [6-8]. Over time, unrecognized defects can progress to chronic herniation, with symptoms appearing months or years after the initial trauma. Cross-sectional imaging, particularly contrast-enhanced computed tomography, is considered the most reliable diagnostic tool, although confirmation may still be required intraoperatively [9,10].

Definitive treatment relies on surgical repair because of the risks of incarceration, strangulation, and progressive respiratory compromise. Minimally invasive approaches, including laparoscopic and robotic techniques, have become increasingly favored in recent years, yet the choice of operative strategy and the use of prosthetic reinforcement remain highly individualized. Evidence regarding long-term outcomes and recurrence is limited, and no consensus-based guidelines currently exist [8,11]. This review aims to synthesize current evidence on the diagnosis, surgical management strategies, and long-term outcomes of chronic post-traumatic diaphragmatic hernias

## Review

Methods

This article was designed as a narrative review with the purpose of consolidating and synthesizing current knowledge regarding chronic post-traumatic diaphragmatic hernias. A narrative review format was selected because the available literature on this topic is highly heterogeneous, encompassing retrospective cohort studies, case series, individual case reports, and expert reviews, which precludes the strict methodology required for a formal systematic review. Unlike systematic reviews that follow structured frameworks such as PRISMA, narrative reviews provide greater flexibility in integrating diverse types of evidence. This approach allows for a more comprehensive exploration of epidemiology, diagnostic pitfalls, surgical strategies, and reported long-term outcomes without attempting to establish definitive treatment protocols.

A comprehensive literature search was conducted in PubMed/MEDLINE, Scopus, Google Scholar, Clinical Key, ResearchGate, Elsevier, DOAJ and Cochrane, covering the period from January 2014 to March 2025. To maximize retrieval, the search strategy combined Medical Subject Headings (MeSH) and free-text terms related to chronic and delayed diaphragmatic hernias. Keywords included “chronic diaphragmatic hernia”, “delayed diaphragmatic hernia”, “acquired diaphragmatic hernia”, “post-traumatic diaphragmatic hernia”, “spontaneous acquired diaphragmatic hernia”, “diagnosis”, “recurrence”, “laparoscopic repair”, “open repair”, and “long-term outcomes”. Boolean operators were applied to optimize retrieval and improve sensitivity, for example:

(“chronic” OR “delayed” OR “acquired”) AND (“diaphragmatic hernia”) NOT (“congenital”),

(“post-traumatic diaphragmatic hernia”) AND (“diagnosis” OR “recurrence” OR “surgical management”),

(“laparoscopic” OR “robotic” OR “minimally invasive”) AND (“diaphragmatic hernia” OR “diaphragmatic rupture”). Reference lists of all included articles were also manually reviewed to identify additional publications not captured by the electronic search.

Inclusion criteria were: (1) studies published between January 2014 and March 2025; (2) peer-reviewed full-text articles in English or Spanish; (3) adult patients (>18 years) with chronic or delayed post-traumatic diaphragmatic hernia; and (4) original research, retrospective or prospective cohort studies, case series, or review articles reporting at least one of the following: epidemiology, diagnostic modalities, clinical presentation, surgical or non-surgical management, recurrence, or long-term outcomes.

Exclusion criteria were: (1) congenital diaphragmatic hernias; (2) single-patient case reports without extractable data; (3) pediatric-only populations; (4) animal or cadaveric studies; (5) abstracts, preprints, conference posters, or non-peer-reviewed materials; and (6) articles without clinical outcomes or insufficient methodological information.

The selection process was conducted in two stages. First, titles and abstracts retrieved from the database search were screened to assess their relevance to CPDH. Articles that did not meet the inclusion criteria, were duplicates, or were clearly unrelated to the topic were excluded at this stage. Second, the full texts of the remaining potentially eligible studies were reviewed in detail. Reference lists of the included studies were also examined manually to identify additional relevant articles not captured by the electronic search. Disagreements regarding study inclusion were resolved through discussion among the reviewers, and inter-rater agreement was calculated, yielding a Cohen’s kappa coefficient of 0.82, indicating substantial agreement.

In total, 32 studies fulfilled the inclusion criteria and were included in the narrative synthesis. However, only eight were true case series with extractable, comparable patient-level data (demographics, diagnostic delays, surgical approach, and outcomes) and were therefore selected for the comparative summary presented in Table 1. This targeted tabulation highlights patterns and differences across the best-defined series, while the broader narrative integrates findings from all 32 publications.

Given the heterogeneity of study designs, patient populations, and outcome reporting, no formal statistical pooling or meta-analysis was performed. Instead, findings were synthesized qualitatively, emphasizing recurring themes, areas of consensus, and divergences across studies. Potential sources of bias-including publication bias, selective reporting, and heterogeneity in study design-were acknowledged as inherent limitations of narrative reviews. Particular attention was given to identifying knowledge gaps and inconsistencies in the literature to provide context for the challenges associated with the diagnosis and management of chronic post-traumatic diaphragmatic hernias.

Results

Diagnosing CPDH remains a clinical challenge due to the frequent absence of specific symptoms in the early stages and the potential for long diagnostic delays. The literature reveals that many patients are initially misdiagnosed or managed under alternative clinical suspicions such as chronic pulmonary disease, gastrointestinal obstruction, or even cardiac symptoms [4,5,11]. The nonspecific nature of symptomsranging from mild dyspnea to acute intestinal obstruction-underscores the need for heightened clinical suspicion, particularly in patients with a history of thoracoabdominal trauma [7,12].

Blunt thoracoabdominal trauma is a leading cause of diaphragmatic rupture, accounting for approximately 35% of acquired cases, while penetrating trauma is responsible for nearly 65% [6,7]. Importantly, up to 31% of patients lack abdominal tenderness at presentation, and nearly 40% may have normal initial chest radiographs, which explains why many acute injuries remain unrecognized and progress to chronic hernias [8]. Over time, these occult defects enlarge, allowing progressive herniation of abdominal viscera into the thoracic cavity, sometimes detected only months or years later [9,10].

Cross-sectional imaging, especially contrast-enhanced CT, is widely considered the diagnostic gold standard [1,13-15]. Nonetheless, several authors have reported that even high-resolution imaging can miss subtle or intermittent herniations, particularly when performed in the acute setting or without proper technique [2,16]. In one retrospective series by Sentürk et al. (2021), over 30% of cases were diagnosed intraoperatively despite prior imaging, highlighting the inherent diagnostic pitfalls and the value of repeat imaging when clinical suspicion persists [8].

Interestingly, pediatric and post-surgical populations present unique diagnostic challenges. In children, the condition may remain occult until adolescence or early adulthood [6]. In post-hepatectomy or posttransplant patients, interpretation of imaging is often confounded by altered anatomical landmarks, making diagnosis particularly difficult [9,13,14].

Surgical repair is universally indicated for CPDH due to the risks of incarceration, strangulation, and respiratory compromise. The traditional approaches-laparotomy and thoracotomy-remain valuable. Laparotomy provides optimal access to intra-abdominal organs and is preferred in cases of ischemia or bowel obstruction [17], whereas thoracotomy facilitates management of dense intrathoracic adhesions [8,18,19,20]. In recent decades, minimally invasive approaches, including laparoscopic and robotic repairs, have gained traction due to reduced morbidity and faster recovery [21,22]. Several series have demonstrated favorable outcomes with laparoscopic repair, even in chronic or recurrent hernias, provided careful patient selection and surgical expertise [10,23,24].

Surgical approach is also influenced by hernia laterality. Right-sided hernias, although less frequent, pose greater technical challenges due to the liver and are associated with higher recurrence rates [2,5]. Left-sided defects are generally more accessible and more commonly repaired laparoscopically [15,25].

Regarding repair techniques, primary tension-free suture closure is feasible for small defects with pliable margins. Larger or chronic hernias often require prosthetic reinforcement to prevent recurrence [23,26]. The debate between synthetic and biological mesh continues. Synthetic meshes remain widely used due to strength and availability, but concerns about infection and erosion persist, particularly in contaminated or emergency settings [25,27]. Biological meshes are increasingly reported as alternatives in these cases, with some studies suggesting lower complication rates, although long-term outcomes and cost-effectiveness remain uncertain [25].

Several case series (including Gu et al., Sentürk et al., Esposito et al., and Sharma et al.) have documented successful outcomes with mesh reinforcement, both via open and minimally invasive techniques [8,12,13,28]. Başol et al. (2022) also showed that laparoscopic approaches are feasible even in complex hernias, though open repair remains necessary in certain contexts [29]. These findings underscore the importance of individualized treatment based on patient characteristics, chronicity, defect size, and surgeon expertise (Table 1).

**Table 1:** Comparative summary of published case series on chronic post-traumatic diaphragmatic hernias (CPDH) This comparative table was constructed based on the information reported in eight case series included in this review. Data regarding patient demographics, hernia characteristics, diagnostic delays, clinical presentation, surgical approach, and postoperative outcomes were extracted directly from the corresponding full-text articles. “Not reported” indicates missing or unavailable data in the original source. M/F: male/female, dx: diagnosis, L/R/B: left/right/bilateral, CT: computed tomography, SOB: shortness of breath, CXR: chest x-ray, CECT: contrast-enhanced computed tomography, MRI: magnetic resonance imaging, SAIO: subacute intestinal obstruction, GSW: gunshot wound, MVC: motor vehicle collision

Author (Year)	No. of Patients	Mean Age (years)	Sex (M/F)	Time from trauma to dx	Symptoms at diagnosis	Affected side (L/R)	Diagnosis method	Surgical approach	Mesh use	Postop complications	Follow-up time	Recurrence
Gu et al. (2019) [12]	19	52 ± 17	8M / 11F	8 ± 7 years	Abdominal pain, nausea, vomiting, hematemesis, chest pain, 3 asymptomatic	14L / 5R	CT	Midline laparotomy, thoracotomy, subcostal laparotomy	No (except 1 acute case)	Intestinal decompression, colostomy	6 ± 4 years	No
Senturk et al. (2021) [8]	20	32.7	13M / 7F	All had trauma (stab/GSW/MVC)	All had trauma; mechanism varied	12L / 6R / 2B	Preop imaging + intraop confirmation	Primary repair (mostly midline), 2 with mesh	Yes (2 cases)	1 death due to organ damage	Not reported	No
Esposito et al. (2017) [14]	3	55	1M / 2F	14 months	Abdominal pain, respiratory symptoms, or both; some asymptomatic	Right	CT	Interrupted suture (2), mesh (1)	Yes (1)	Not specified	1, 5, 10 months	No
Cortes et al. (2014) [13]	10	4.9	5M / 5F	3.8 years	Not specified	Right	X-ray + CT	Not specified	Yes (3)	Not specified	Not reported	Not specified
Başol et al. (2022) [29]	22	54	6M / 16F	Not reported	Abdominal pain, SOB, early satiety, nausea, vomiting, distension	90% Right	X-ray, CT, contrast studies	Open (7), Laparoscopic (15)	No	Not reported	Not specified	No
Lamture et al. (2024) [11]	2	48	1M / 1F	10d/2d	Left hypochondrial pain (F), diffuse pain (M)	Left	CXR + CECT	Open with composite mesh	Yes	None	1 month	No
Sharma et al. (2025) [4]	3	43	2M / 1F	1-3 years	SAIO, vomiting, dyspnea, constipation	Left	CT	Laparoscopic hernioplasty	Yes	Not available	Not available	Not available
Liu et al. (2021) [10]	23	53	16M / 7F	Not reported	Nonspecific; chest pain, dyspnea, nausea, vomiting, fever	90% Left	CT, MRI, GI studies	Laparoscopic only	Yes (large defects ≥5 cm)	Mild (pleural effusion, fever, pain)	1–50 months	Not available

Postoperative outcomes are generally favorable when repair is performed electively by experienced teams. Nonetheless, complication rates remain significant, especially in delayed or emergency cases. Common postoperative issues include pleural effusion, pneumonia, wound infection, and prolonged ileus [17,30]. Recurrence rates vary widely across series, ranging from 0% to more than 20%, depending on defect size, repair method, and mesh use [4,23]. Recurrence is particularly associated with large or chronic defects closed primarily without prosthetic support [25,31]. Right-sided hernias and urgent repairs are also linked to higher recurrence and complication rates [2,5].

In the subset of iatrogenic hernias following liver surgery or esophagectomy, outcomes largely depend on early recognition and the integrity of the repair [1,9,32]. Close follow-up and timely imaging in high-risk populations may help reduce long-term morbidity.

Despite the growing body of literature on CPDH, the evidence remains fragmented and largely limited to retrospective case series and small institutional experiences. The heterogeneity in study designs, patient populations, and reporting standards makes direct comparison difficult and precludes robust conclusions regarding optimal surgical techniques or the superiority of specific mesh materials. Notably, recurrence appears to be minimized with prosthetic reinforcement, yet long-term outcomes are inconsistently reported and often limited to short follow-up periods. The predominance of left-sided cases in the literature also raises the possibility of underreporting or underrecognition of right-sided hernias, which may carry distinct diagnostic and technical challenges. Moreover, the frequent reliance on intraoperative diagnosis highlights ongoing limitations in imaging accuracy, underscoring the need for standardized diagnostic protocols. Future studies should aim to provide multicenter, prospective data with standardized definitions, detailed reporting of surgical strategies, and long-term follow-up to better inform evidence-based guidelines for the management of CPDH.

In summary, although the evidence on chronic post-traumatic diaphragmatic hernias remains largely limited to retrospective series and institutional experiences, recent high-quality studies have begun to bridge this gap. Large trauma database analyses, such as that by Fair et al. [33], have provided a broader epidemiologic perspective on diaphragmatic injuries, while contemporary meta-analyses and comparative studies by Major et al. [28] and Young et al. [30] have strengthened the evidence supporting minimally invasive repair in appropriately selected cases. Together, these findings underscore the progressive shift toward evidence-based, standardized management of CPDH, highlighting the importance of multicenter collaboration and prospective data collection to refine surgical strategies and improve long-term outcomes.

## Conclusions

CPDHs continue to represent a diagnostic and therapeutic challenge due to their often delayed presentation and nonspecific clinical manifestations. Although contrast-enhanced CT remains the most reliable diagnostic tool, a significant proportion of cases are still identified intraoperatively, underscoring the ongoing limitations of imaging modalities. Surgical repair remains the definitive treatment, with minimally invasive techniques, particularly laparoscopic and robotic approaches, demonstrating lower morbidity and faster recovery in well-selected patients. The choice between primary closure and mesh reinforcement should be individualized according to defect size, chronicity, and contamination risk, as prosthetic reinforcement has shown reduced recurrence rates in larger or chronic defects.

Despite advancements in diagnosis and minimally invasive repair, the evidence base for CPDH management remains fragmented, largely derived from small retrospective series. Future research should focus on multicenter prospective studies to standardize diagnostic algorithms, clarify optimal repair techniques, and evaluate long-term outcomes and recurrence. Integration of large trauma registries and collaborative surgical databases, as suggested by recent systematic reviews, will be essential to establish evidence-based guidelines and improve both clinical decision-making and patient prognosis.
